# Spermidine protects against acute kidney injury by modulating macrophage NLRP3 inflammasome activation and mitochondrial respiration in an eIF5A hypusination-related pathway

**DOI:** 10.1186/s10020-022-00533-1

**Published:** 2022-09-04

**Authors:** Xianzhi Li, Xiaojun Zhou, Xigao Liu, Xiaoyun Li, Xianzhou Jiang, Benkang Shi, Shuo Wang

**Affiliations:** 1 Department of Urology, Qilu Hospital of Shandong University, 107 Wenhua Xi Road, Jinan, 250012 Shandong China; 2 Department of Endocrinology and Metabology, The First Affiliated Hospital of Shandong First Medical University & Shandong Provincial Qianfoshan Hospital, Shandong Key Laboratory of Rheumatic Disease and Translational Medicine, Shandong Institute of Nephrology, Jinan, 250014 China; 3 Department of Otolaryngology, Qingdao Eighth People’s Hospital, Qingdao, 266121 China

**Keywords:** Spermidine, Polyamine, Acute kidney injury, Macrophage, NLRP3

## Abstract

**Background:**

Acute kidney injury (AKI) is still a critical problem in clinical practice, with a heavy burden for national health system around the world. It is notable that sepsis is the predominant cause of AKI for patients in the intensive care unit and the mortality remains considerably high. The treatment for AKI relies on supportive therapies and almost no specific treatment is currently available. Spermidine is a naturally occurring polyamine with pleiotropic effects. However, the renoprotective effect of spermidine and the underlying mechanism remain elusive.

**Methods:**

We employed mice sepsis-induced AKI model and explored the potential renoprotective effect of spermidine in vivo with different administration time and routes. Macrophage depleting was utilized to probe the role of macrophage. In vitro experiments were conducted to examine the effect of spermidine on macrophage cytokine secretion, NLRP3 inflammasome activation and mitochondrial respiration.

**Results:**

We confirmed that spermidine improves AKI with different administration time and routes and that macrophages serves as an essential mediator in this protective effect. Meanwhile, spermidine downregulates NOD-like receptor protein 3 (NLRP3) inflammasome activation and IL-1 beta production in macrophages directly. Mechanically, spermidine enhances mitochondrial respiration capacity and maintains mitochondria function which contribute to the NLRP3 inhibition. Importantly, we showed that eukaryotic initiation factor 5A (eIF5A) hypusination plays an important role in regulating macrophage bioactivity.

**Conclusions:**

Spermidine administration practically protects against sepsis-induced AKI in mice and macrophages serve as an essential mediator in this protective effect. Our study identifies spermidine as a promising pharmacologic approach to prevent AKI.

**Supplementary Information:**

The online version contains supplementary material available at 10.1186/s10020-022-00533-1.

## Introduction

Acute kidney injury (AKI), marked by a sudden loss of kidney function, is a common and severe clinical condition with high morbidity, mortality and hospitalization costs (Zuk and Bonventre 2016). Nowadays, the global burden of AKI remains high (Mehta et al. 2015; Raimann et al. 2018). The yearly incidence of AKI (6800 per million population) has been estimated to exceed that of myocardial infarction (Kellum et al. 2018). The distribution of causes of AKI differs with the settings in which it occurs. Of note, sepsis is the predominant cause of AKI for patients in the intensive care unit and accounts for 45–70% of all AKI cases (Sun et al. 2019; Peerapornratana et al. 2019). Management of AKI essentially relies on supportive modalities, including renal replacement therapy, fluid and electrolyte management, and almost no specific therapeutics are currently available for renal damages (Kellum et al. 2021). Therefore, development of novel therapeutic interventions for AKI is urgently needed.

Spermidine is a naturally occurring polyamine that is indispensable for cell growth and proliferation (Partridge et al. 2020). Increasing evidence have proved that spermidine can exert lifespan-extending effect on different living organisms (Eisenberg et al. 2009, 2016; Yue et al. 2017). A prospective population-based study in human exhibited a positive correlation between dietary spermidine intake and decreased all-cause mortality (Kiechl et al. 2018). Importantly, it has also been implicated to provide protection to different tissues and organs, including but not limited to brain, heart, liver and kidney (Madeo et al. 2018; Gupta et al. 2016; Liu et al. 2019; Okumura et al. 2016; Chai et al. 2019). The safety concerns of pharmacological candidates are indeed critical issues in clinical trials. Given that spermidine is ubiquitous in cells across species and commonly present in food and human nutrition, spermidine seems well-tolerable as a therapeutic drug.

Despite previous reports on the positive effects of spermidine for kidney injuries, the underlying mechanism are not fully understood and warrant further investigations. In the present study, we first confirmed the potential renoprotection of spermidine in mouse model of sepsis-induced AKI. We found that the protective effect of spermidine was largely abolished when macrophages were pharmacologically depleted. Furthermore, we demonstrated that spermidine inhibits macrophage NOD-like receptor protein 3 (NLRP3) inflammasome activation and improves mitochondrial dysfunction in a eukaryotic initiation factor 5A (eIF5A) hypusination-dependent pathway. Our study extends the previous understanding of the pleiotropic effects of spermidine.

## Materials and methods

### Mouse model of sepsis-induced AKI

**Fig. 1:**
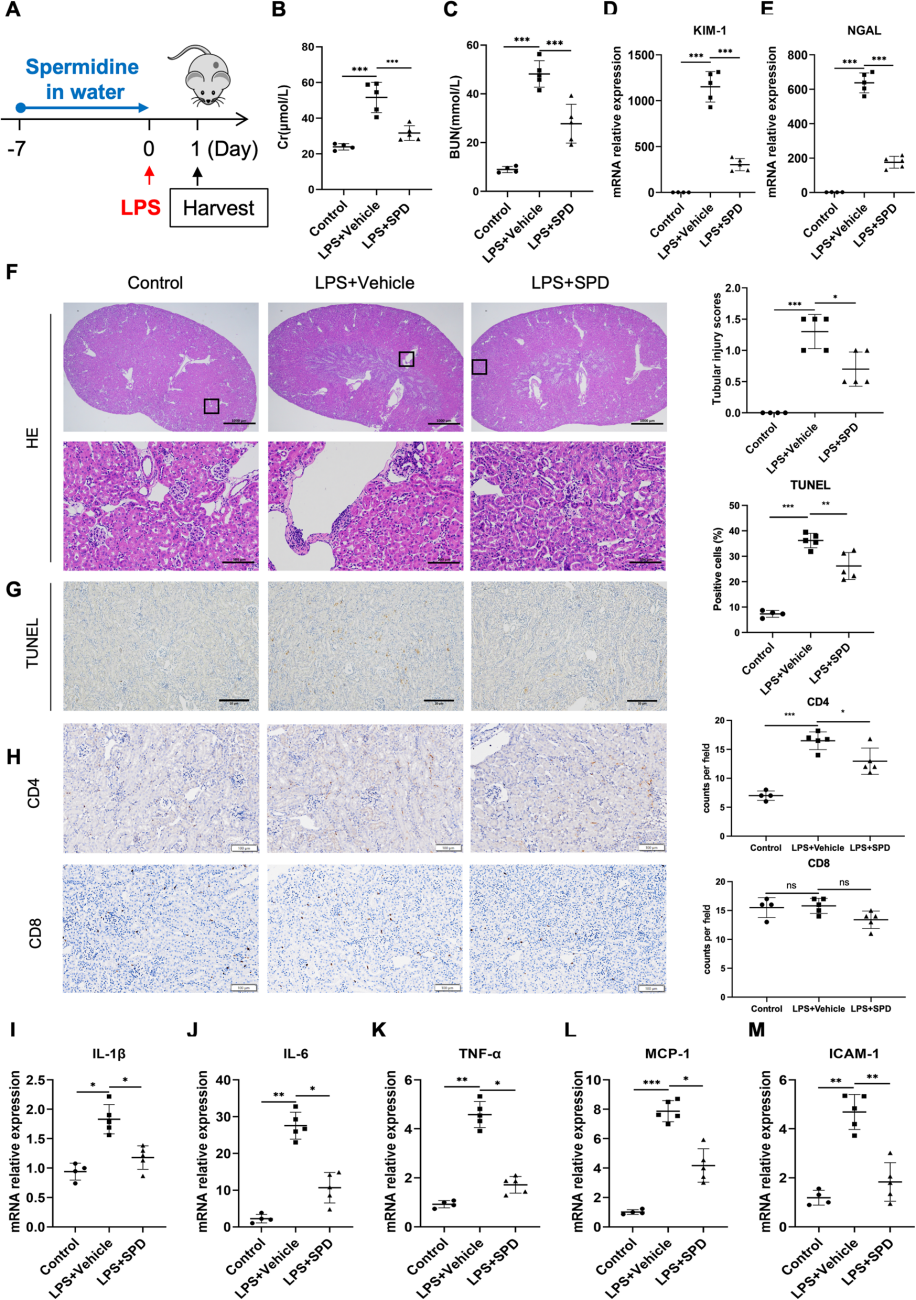
Spermidine supplementation prior to AKI prevents renal damages and inflammatory. **A** Schematic overview of spermidine treatment to C57/BL6 mice. Spermidine (SPD) was administered into drinking water (3 mM) for 1 week before sepsis induction. Mice were subjected to LPS intraperitoneally at a dose of 10 mg/kg. Twenty-four hours after LPS injection, mice were sacrificed for blood and kidney samples. Levels of **B** serum creatinine (Cr) and **C** blood urea nitrogen (BUN) were detected. n = 4–5 mice per group. Renal mRNA levels of **D** KIM-1, **E** NGAL were analyzed by qPCR. **F** Representative images of HE staining are exhibited, and tubular injury scores were calculated. Scale bar, 1 mm; inset scale bar, 100 μm. **G** Representative photographs of TUNEL staining. Quantitative analysis of TUNEL-positive cells was performed. Scale bar, 20 μm. **H** Immunohistochemical staining of CD4 and CD8 in kidney tissue. Scale bar, 100 μm. Renal mRNA levels of **I** IL-1β, **J** IL-6, **K** TNF-α, **L** MCP-1, and **M** ICAM-1 were analyzed by qPCR. Data are expressed as mean ± SEM. One-way ANOVA followed by post hoc Sidak test. *P < 0.05, **P < 0.01, ***P < 0.001

**Fig. 2:**
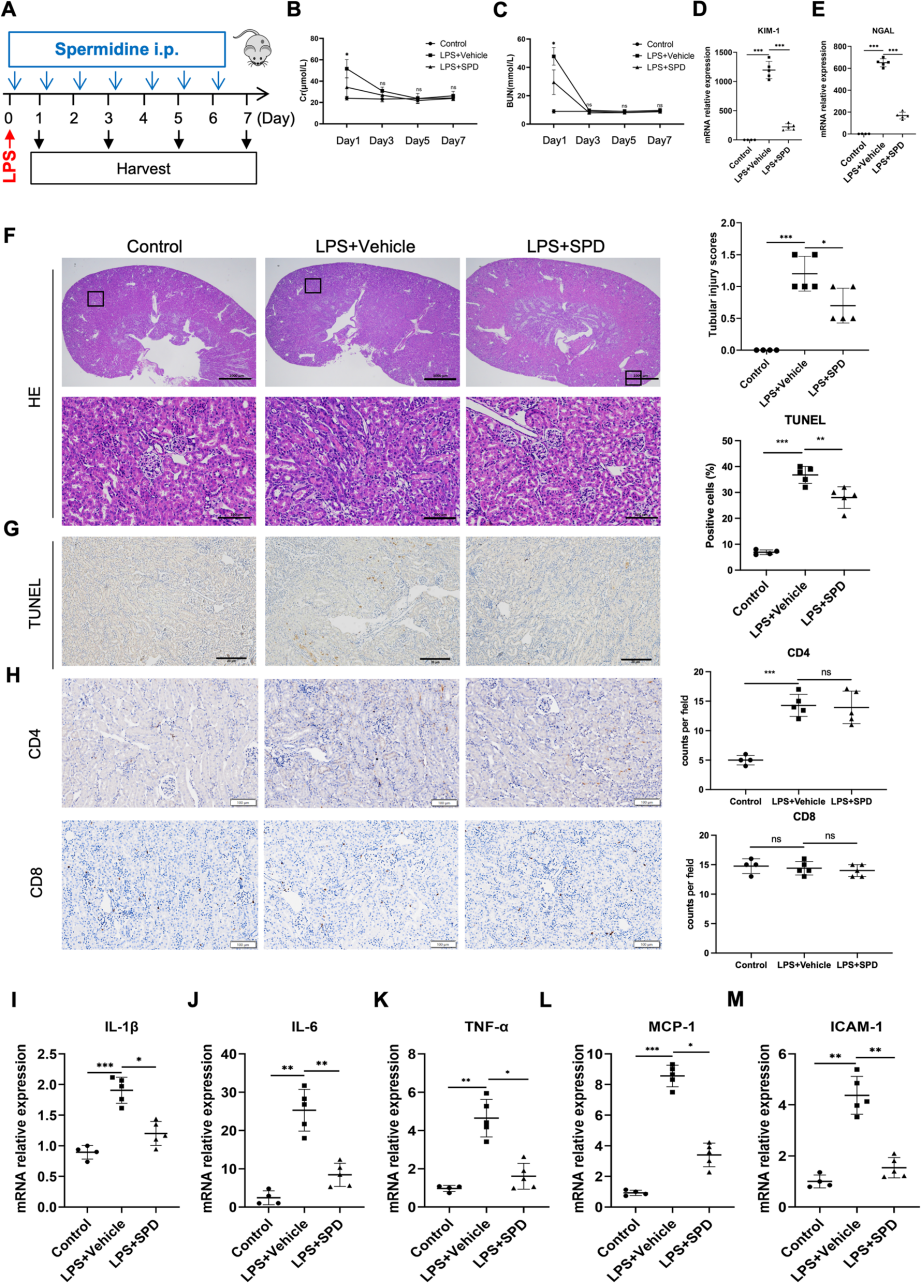
Spermidine administration following LPS injection attenuates kidney impairments and inflammation. **A** Mice were subjected to LPS intraperitoneally at a dose of 10 mg/kg. Spermidine (50 mg/kg) was administered intraperitoneally (i.p.) daily 30 min after sepsis induction. Mice were sacrificed for blood and kidney samples at indicated time points. Levels of **B** serum creatinine (Cr) and **C** blood urea nitrogen (BUN) were detected. *P < 0.05 LPS + Vehicle vs LPS + SPD. Control, n = 4 per time point; other groups, n = 5 per time point. Renal mRNA levels of **D** KIM-1 and **E** NGAL on day 1 were analyzed by qPCR. **F** Representative images of HE staining on day 1 are exhibited, and tubular injury scores were calculated. Scale bar, 1 mm; inset scale bar, 100 μm. n = 3. **G** Representative photographs of TUNEL staining on day 1. Quantitative analysis of TUNEL-positive cells was performed. Scale bar, 20 μm. **H** Immunohistochemical staining of CD4 and CD8 in kidney tissue. Scale bar, 100 μm. Renal mRNA levels of **I** IL-1β, **J** IL-6, **K** TNF-α, **L** MCP-1, and **M** ICAM-1 on day 1 were analyzed by qPCR. Data are expressed as mean ± SEM. One-way ANOVA followed by post hoc Sidak test. *P < 0.05, **P < 0.01, ***P < 0.001

All animal experiments were approved by the ethical committee of Qilu Hospital of Shandong University. Male C57BL/6 mice with 6–8 weeks of age (20–25 g in weight) were purchased from Jinan Pengyue Bio-Technology Co. Ltd. (Jinan, China), housed in a pathogen-free, temperature-controlled environment under a 12-h light/dark cycle, and had free access to food and water. To induce sepsis-associated AKI, mice were subjected to a single dose of 10 mg/kg LPS (Sigma-Aldrich, St Louis, MO, USA) intraperitoneally (i.p.). Mice were randomly divided into three cohorts with different administration time and routes of spermidine (SPD, S0266, Sigma-Aldrich, St Louis, MO, USA). There were three groups in each cohort: control group, LPS group and SPD group (n = 4, control group; n = 5, LPS group and SPD group at each time point). SPD group in cohort 1 received spermidine (3 mM) through drinking water (Fig. 1A). The oral supplementation started from 1 week before LPS treatment, on the basis of our preliminary data (Additional file 1: Fig. S1A). The dose of spermidine was selected based on previous studies and dose–response experiments (Additional file 1: Fig. S1B). At this dose (3 mM), spermidine has been shown to have positive effects in different pathological settings, including aging, abdominal aortic aneurysms, and liver fibrosis (Eisenberg et al. 2016; Liu et al. 2019, 2020b). Water consumption was recorded daily, and the drinking water was changed every 2 days. SPD group in cohort 2 received daily intraperitoneal injection of spermidine (50 mg/kg body weight) 30 min post LPS treatment (Fig. 2A). SPD group in cohort 3 received daily intraperitoneal injection of spermidine (50 mg/kg body weight) 24 h after LPS treatment (Additional file 1: Fig. S2A). Mice were sacrificed at indicated time points. Blood samples and kidneys were collected for measurements performed in the study.

### Macrophage depletion

To selectively deplete macrophages, male C57BL/6 mice were intraperitoneally injected with 200 μl of clodronate liposomes (CL) or empty liposomes (EL, Dakewe, Beijing, China) according to the manufacturer’s guidance. LPS injection was performed 24 h after depletion. Mice were sacrificed to obtain samples on day 1 following LPS injection. Mice were randomly divided into five groups. Due to mice death, before harvesting of tissue and blood, six mice remained in control group and EL + LPS + Vehicle group, seven in EL + LPS + SPD group, five in CL + LPS + Vehicle group and CL + LPS + SPD group.

### Detection of kidney function

Whole-blood samples were obtained via cardiac puncture and allowed to clot at room temperature, followed by centrifugation for collection of serum. Serum creatinine (Cr) and blood urea nitrogen (BUN) were measured as indicators of kidney function using a Chemray 800 Automatic biochemical analyzer (Radu Life Sciences Ltd, Japan).

### Histologic analysis

To evaluate histological injury, 4% paraformaldehyde-fixed and paraffin-embedded kidney blocks were cut into 3-μm sections and then subjected to standard hematoxylin and eosin (HE) staining. Tissue sections were viewed by two experienced pathologists blinded to the treatment group. At least eight randomly selected regions of the corticomedullary junction from each mouse was measured. The histological alterations were calculated as tubular injury score, which was quantified on a 0–4 scale according to the percentage of tubular necrosis, tubular dilation and cast formation (0, none; 1, 1–25%; 2, 26–50%; 3, 51–75%; 4, > 75%).

### TdT mediated dUTP nick end labeling (TUNEL) assay

To detect apoptosis of kidney tissues, TUNEL assay was performed using a DAB (SA-HRP) TUNEL Cell Apoptosis Detection Kit (Servicebio, Wuhan, China) according to the manufacturer’s instruction. At least eight randomly selected areas from each mouse of different groups were assessed under light microscope. The percentage of TUNEL-positive cells was calculated using ImageJ software (National Institutes of Health, Bethesda, MD, USA).

### Immunohistochemistry

Immunohistochemical staining was performed according to a routine procedure. Briefly, paraffin-embedded kidney sections were rehydrated through graded ethanol series and incubated in 0.3% hydrogen peroxide to block the endogenous peroxidase activity. For detection of F4/80, CD4 and CD8α, sections were incubated with the primary antibodies overnight at 4 °C, followed by incubation with horseradish peroxidase-labeled goat anti-rabbit IgG secondary antibody (Zhongshan Jinqiao Biotechnology Co., Ltd, Beijing, China) for 1 h at room temperature. The primary antibodies are shown as follows: F4/80, rabbit anti-mouse, 1:200 (CST, Danvers, MA, USA); CD4, rabbit anti-mouse, 1:500 (Abcam, Cambridge, MA, USA); CD8α, rabbit anti-mouse, 1:200 (Abcam). The specimens were then stained with diaminobenzidine (DAB) and counterstained with hematoxylin. ImageJ software was used for quantitative image analysis.

### Cell culture and treatment

THP-1 cell line was obtained from Cell Bank of Chinese Academy of Science and cultured in RPMI-1640 medium (Corning, NY, USA) supplemented with 10% fetal bovine serum (FBS) (Lonsera, Uruguay), 100 units/ml penicillin and 100 mg/ml streptomycin (Gibco, Shanghai, China). In all experiments, THP-1 cells were cultured in 6-well plates and treated with 100 ng/ml of 12-myristate 13-acetate (PMA; Sigma-Aldrich, USA) for 48 h to transform into the resting state of macrophages. To obtain primary peritoneal macrophage, mice were treated intraperitoneally with 4% thioglycollate (Sigma-Aldrich) for 4 days. Peritoneal exudate cells were collected by peritoneal lavage and culture in complete medium overnight. Before the stimulations, nonadherent cells were removed by washing with PBS. Macrophages were pretreated with fresh medium supplemented with 50 μM of spermidine for 1 h, followed by administration of 200 ng/ml LPS for 24 h.

### Enzyme-linked immunosorbent assay (ELISA)

The concentrations of interleukin-1β (IL-1β), interleukin-6 (IL-6) and monocyte chemoattractant protein-1 (MCP-1) in the supernatant of cell culture were determined by ELISA kits (Boster Biological Technology, Wuhan, China) according to the manufacturer’s instructions.

### Quantitative real-time polymerase chain reaction (qPCR)

Total RNA was isolated from kidney tissues and cultured cells by TRIzol reagent (Invitrogen, CA, USA). Reverse transcription was carried out by a FastKing one-step reverse transcription reagent kit (Tiangen, Beijing, China). Quantitative RT-PCR (qRT-PCR) was performed using SYBR Green master mix (TaKaRa, Dalian, China) according to the manufacturer’s protocol. Gene expression levels were presented as fold exchange that was normalized to β-actin in control group. Primer sequences are listed in Additional file 2: Table S1.

### High-resolution respirometry

Mitochondria respiration and mitochondrial membrane potential of macrophages were analyzed at 37 °C using Oxygraph-2k (O2k) high-resolution respirometer (Oroboros Instruments, Innsbruck, Austria) according to the manufacturer’s instruction. Cultured THP-1 cells were collected, washed, and then resuspended in respiration buffer at a final concentration of 0.5–1 × 10^6^ cells/ml. The number of cells loaded into the chamber (1 × 10^6^ cells) served as an estimate of mitochondrial amount used for normalization. Oxygen consumption rates were measured with substrates and inhibitors in the following sequence: malate (5 mM), ADP (2 mM), glutamate (5 mM), succinate (5 mM), rotenone (1 mM), oligomycin (1.25 μM), CCCP (0.5 μM), antimycin A (0.5 μM). Digitonin was used for permeabilization.

### Reactive oxygen species (ROS) and mitochondrial membrane potential detection

To examine mitochondrial ROS, THP-1 macrophages were incubated with MitoSOX™ Red reagent working solution (Molecular Probes, USA) for 10 min at 37 °C, followed by staining with Hoechst 33258. Subsequently, the cells were observed with a fluorescence microscope (Olympus FSX100).

Mitochondrial membrane potential assay kit (JC-1) (Beyotime, Shanghai, China) was used to detect the mitochondrial membrane potential of THP-1 cells. Cells were washed and then stained with JC-1 solution for 20 min at 37 °C in the dark. THP-1 cells were washed with PBS and observed under a fluorescence microscope. The relative aggregate-to-monomer (red/green) fluorescence intensity ratio was used as an indicator of the alterations of mitochondrial membrane potential.

### Western blotting

Western blotting analysis was performed as previously described (Wang et al. 2017). Briefly, total proteins from cultured cells were obtained, separated by SDS–PAGE electrophoresis and transferred onto nitrocellulose membranes. Primary antibodies against IL-1β (1:1000, CST), NLRP3 (1:500, Proteintech), NF-κB (1:1000, CST), p-NF-κB (1:1000, CST), Atp5a1 (1:2000, Proteintech), Ndufv2 (1:5000, Proteintech), β-actin (1:10,000, Proteintech) and appropriate secondary antibodies were used. The optical density of bands was analyzed by ImageJ software. The expression levels of the examined proteins were normalized to those of β-actin.

### Statistical analysis

Statistical analyses were performed using Graph Prism 8.0 (GraphPad Software, Inc). For in vivo experiments, n = number of individual animals. For in vitro experiments, n = number of biological replicates. Multiple statistic comparisons were analyzed using one-way or two-way ANOVA followed by Sidak’s multiple comparisons test. Data are expressed as the means ± S.E.M., and differences were deemed significant when *P* < 0.05.

## Results

### Oral supplementation of spermidine prior to AKI prevents renal damages and inflammatory responses

To explore the potential renoprotective effect of spermidine, an established mouse model of sepsis-induced AKI was performed with LPS. Spermidine was administered to mice in drinking water (3 mM) for 7 days before LPS injection (Fig. 1A). Average water consumption (4 ml/mouse/day) and body weight of mice in different groups were similar. As expected, LPS resulted in obvious renal dysfunction in mice, demonstrated by elevated serum Cr and BUN levels. By contrast, 1 week of oral supplementation of spermidine successfully reduced Cr and BUN levels in response to LPS insults (Fig. 1B, C). In addition, renal mRNA expressions of kidney injury molecule-1 (KIM-1) and neutrophil gelatinase-associated lipocalin (NGAL), both of which are kidney injury biomarkers, were lower in spermidine-treated mice relative to those of LPS group (Fig. 1D, E). Renal pathological evaluation was performed based on HE staining and quantitatively calculated as tubular injury score. Meanwhile, TUNEL assay was conducted to detect tissue apoptosis in situ. As shown in Fig. 1F and G, spermidine improved tubular necrosis, tubular dilatation and cast formation accompanied by decreased tubular injury scores, and diminished renal apoptosis. We also determined the infiltration of CD4^+^ and CD8^+^ T cells through immunohistochemical staining and found that the number of CD4^+^ T cells in kidney tissue decreased in spermidine-treated group (Fig. 1H). Moreover, renal expression of proinflammatory factors were further examined by real-time qPCR to evaluate the inflammatory status in kidneys. Parallel to functional and structural results, the PCR analysis revealed that spermidine significantly decreased expressions of proinflammatory cytokines including IL-1β, IL-6, tumor necrosis factor-α (TNF-α), chemokine MCP-1 and adhesion molecule intercellular cell adhesion molecule-1 (ICAM-1) (Fig. 1I–M), indicating that spermidine efficiently prevents kidney impairments as well as renal inflammation.

### Spermidine treatment following LPS injection attenuates kidney impairments and inflammation

We then wondered whether administration of spermidine following LPS injection could still exert a therapeutic effect. For this purpose, intraperitoneal route was adopted to accurately manage the treatment dosage of spermidine. Spermidine was administered daily via intraperitoneal injection at a dose of 50 mg/kg body weight from 30 min after LPS treatment (Fig. 2A). Both Cr and BUN peaked on day 1, then decreased thereafter, completely recovered on day 5 (Fig. 2B, C). Consistent with previous results, intraperitoneal addition of spermidine significantly reduced the levels of Cr, BUN, KIM-1 and NGAL (Fig. 2B–E), improved histological damages (Fig. 2F), attenuated apoptosis (Fig. 2G) on day 1. However, spermidine failed to inhibit the infiltration of CD4^+^ T cells on day 1 (Fig. 2H). Additionally, spermidine also markedly reversed the LPS-induced upregulation of renal injury markers and inflammatory molecules (Fig. 2I–M).

We next investigated the effect of spermidine with treatment from 24 h post LPS injection (Additional file 1: Fig. S2A). The results showed that serum levels of Cr and BUN, as well as histological damage were significantly decreased in SPD group on day 2 (Additional file 1: Fig. S2B–D). Collectively, these results suggested that spermidine could serve as a potent therapeutic agent for AKI with diverse timing and routes of administration.

### Macrophage participates in the renoprotective effects of spermidine

**Fig. 3:**
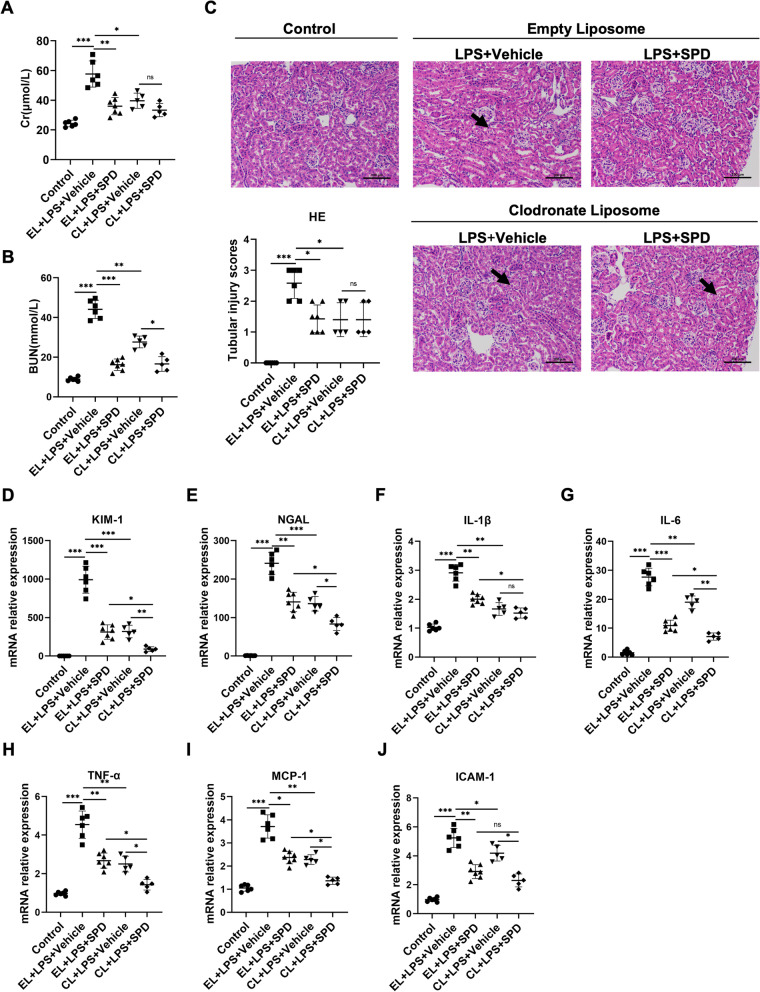
The renoprotection of spermidine largely relies on macrophage. Clodronate liposome (CL) was administered intravenously 24 h before LPS injection to deplete macrophages. Empty liposome (EL) was used as control. Spermidine was administered intraperitoneally following LPS injection. Control n = 6, EL + LPS + Vehicle n = 6, EL + LPS + SPD n = 7, CL + LPS + Vehicle n = 5, CL + LPS + SPD n = 5. Levels of **A** serum creatinine (Cr) and **B** blood urea nitrogen (BUN) were detected. **C** Representative images of HE staining are exhibited, and tubular injury scores were calculated. Black arrow indicated the tubular vacuolation. Scale bar, 100 μm. Renal mRNA levels of **D** KIM-1, **E** NGAL, **F** IL-1β, **G** IL-6, **H** TNF-α, **I** MCP-1, and **J** ICAM-1 were analyzed by qPCR. Data are expressed as mean ± SEM. Two-way ANOVA followed by post hoc Sidak test. *P < 0.05, **P < 0.01, ***P < 0.001

Macrophage plays an important role in the pathophysiology of AKI (Jang and Rabb 2015; Rogers et al. 2014; Weisheit et al. 2015). Previous study has demonstrated spermidine could alleviate experimental autoimmune encephalomyelitis by modulating macrophage polarization (Yang et al. 2016). To probe the role of macrophage in the renoprotection of spermidine, clodronate liposomes (CL), a macrophage-depleting drug, were administered intraperitoneally to mice 24 h before the LPS injection. The same volume of empty liposomes (EL) suspension was used as control. Macrophage reduction by clodronate was confirmed by immunohistochemical detection (Additional file 1: Fig. S3). Consistent with previous report (Ferenbach et al. 2012), macrophage depletion exerted a beneficial effect on AKI (Fig. 3A–J, compare *CL* + *LPS* + *Vehicle* to *EL* + *LPS* + *Vehicle*). However, spermidine offered no additional therapeutic effect in combined treatment with clodronate liposomes in terms of Cr level and pathological damage (Fig. 3A and C, compare *CL* + *LPS* + *SPD* to *CL* + *LPS* + *Vehicle*). Similarly, macrophage depleting largely diminished the effect of spermidine to lower BUN level (Fig. 3B). These results indicated that macrophage is an important, although perhaps not exclusive, contributor to the renoprotective effects of spermidine. In support of this concept, clodronate liposomes also obviously abrogated the inhibition of spermidine for renal expressions of renal injury markers and proinflammatory factors (Fig. 3D–J).

To clarify whether spermidine improves AKI by simply modulating macrophages recruitment, the dynamics of renal infiltration of macrophages was explored. Immunohistochemical analysis for the pan-macrophage marker F4/80 showed a trend of decreasing the number of macrophages by spermidine (Additional file 1: Fig. S4). However, the inhibition for macrophage infiltration reached a statistically significance only on day 5. Contrastingly, we previously showed that spermidine exerted its therapeutic effect at the early stage of AKI. These data therefore excluded the speculation that spermidine might offer renal protection by downregulating macrophages infiltrations.

### Spermidine suppresses LPS-induced NLRP3 inflammasome activation and IL-1β production in macrophages

**Fig. 4:**
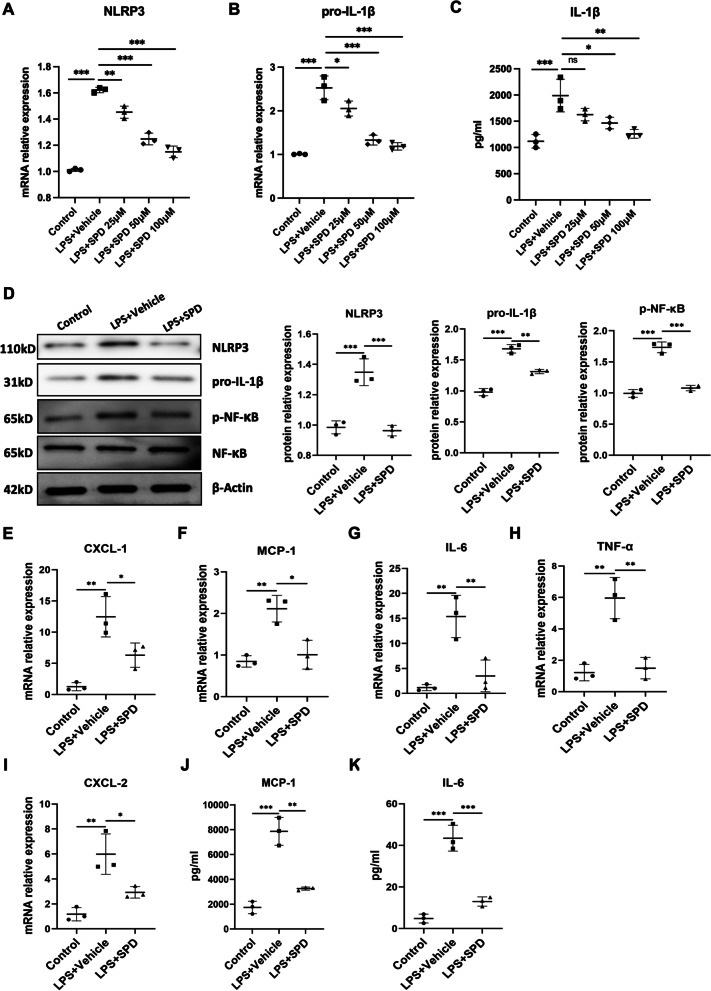
Spermidine inhibits macrophage NLRP3 inflammasome activation and IL-1β production. PMA-differentiated THP-1 macrophages were pretreated with spermidine for 1 h and then stimulated by LPS (200 ng/ml) for 24 h. **A** NLRP3 and **B** pro-IL-1β mRNA expression were detected by qPCR. **C** Secretion of IL-1β was determined by ELISA. **D** THP-1 macrophages were pretreated with spermidine (50 μM) for 1 h and then stimulated by LPS (200 ng/ml) for 24 h. Western blot analysis of NLRP3, pro-IL-1β, p-NF-κB, NF-κB and β-actin. Cellular mRNA levels of **E** CXCL-1, **F** MCP-1, **G** IL-6, **H** TNF-α, **I** CXCL-2 were examined by qPCR. Levels of **J** MCP-1 and **K** IL-6 in the culture supernatants were determined by ELISA. Experiments were performed in triplicate. Data are expressed as mean ± SEM. One-way ANOVA followed by post hoc Sidak test. *P < 0.05, **P < 0.01, ***P < 0.001

NLRP3 plays a key role to initiate immune response in macrophage under cellular stress by processing pro-inflammatory cytokines such as IL-1β. Previous studies have demonstrated that spermidine inhibits the inflammatory responses of macrophages (Yang et al. 2016; Liu et al. 2020a). Based on previous observations, we here further explored the effect of spermidine on NLRP3 inflammasome activation. Human macrophage THP-1 cells were pretreated with spermidine, and then stimulated with LPS. Previous study has shown that LPS was sufficient to induce NLRP3 activation and IL-1β secretion even in the absence of second signals (for example, ATP) (Ip et al. 2017). Consistently, we confirmed a significant upregulation of NLRP3 and pro-IL-1β expression in LPS-treated THP-1 cells (Fig. 4A–D). Importantly, we found that spermidine inhibited LPS-induced NLRP3 and IL-1β expression in a dose-dependent manner (Fig. 4A–C). Western blotting also demonstrated pretreatment with spermidine markedly decreased protein levels of NLRP3 and pro-IL-1β (Fig. 4D). Given that engagement of NF-κB is necessary for NLRP3 and pro-IL-1β pathway (Franchi et al. 2012), we next determined whether spermidine regulates LPS-induced NF-κB activation. Our data revealed that spermidine significantly impaired NF-κB phosphorylation induced by LPS (Fig. 4D). As expected, spermidine also attenuated the synthesis and secretion of several other inflammatory cytokines (IL-6, TNF-α) and chemokines (CXCL-1, CXCL-2, MCP-1) in LPS-stimulated THP-1 macrophages (Fig. 4E–K). Collectively, our findings here highlight an important role for spermidine in modulating NLRP3 inflammasome activation, by which spermidine restrains the pro-inflammatory capacities of macrophages.

### Spermidine augments mitochondrial respiration and maintains mitochondrial fitness of macrophages in response to LPS stimulation

**Fig. 5:**
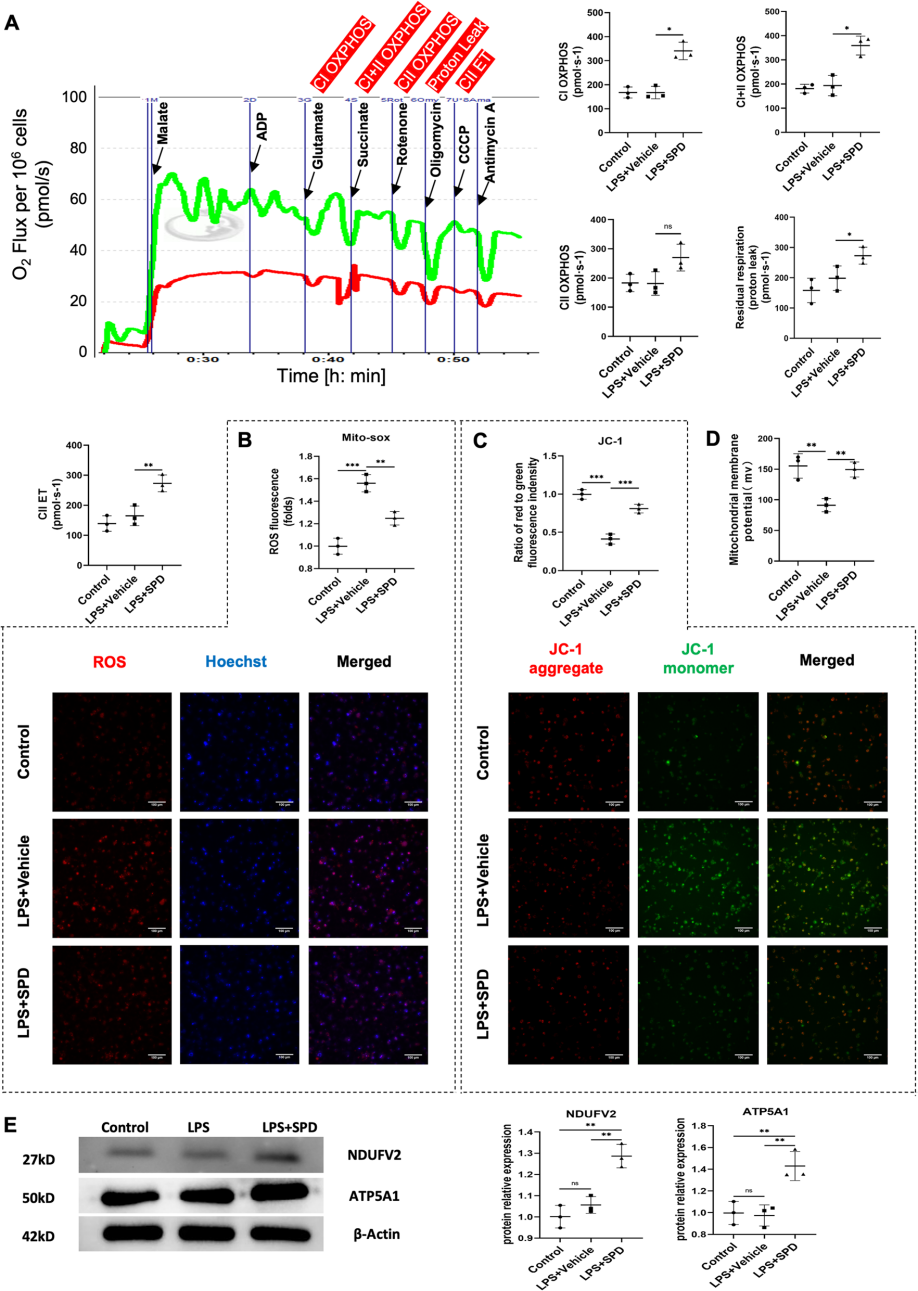
Spermidine promotes mitochondrial respiration and prevents mitochondrial dysfunction. **A** Oxygen consumption measurements of THP-1 macrophages by high-resolution respirometer. Digitonin was used for permeabilization. Malate (5 mM), ADP (2 mM) and glutamate (5 mM) to measure complex I-driven phosphorylating respiration (CI OXPHOS); succinate (5 mM) to measure CI + II OXPHOS; rotenone (1 mM) to inhibit complex I-driven respiration and measure CII OXPHOS; oligomycin (1.25 μM) to measure residual respiration (proton leak); CCCP (0.5 μM) to measure complex II-driven maximal uncoupled respiration (CII electron transfer capacity, CII ET). **B** ROS immunostaining (red) and Hoechst (blue) staining. Scale bar, 100 μm. Fluorescence intensity was calculated using Image J software. **C** JC-1 staining. The ratio of aggregate-to-monomer (red/green) fluorescence intensity represents mitochondrial membrane potential and was quantified using Image J software. Scale bar, 100 μm. **D** Mitochondrial membrane potential probed by O2k respirometer. **E** Western blot analysis of NDUFV2 and ATP5A1. Experiments were performed in triplicate. Data are expressed as mean ± SEM. One-way ANOVA followed by post hoc Sidak test. *P < 0.05, **P < 0.01, ***P < 0.001

Damaged, ROS-generating mitochondria was shown to be needed for NLRP3 inflammasome activation (Zhou et al. 2011; Zhong et al. 2018). Due to the known protection of spermidine for mitochondria, we therefore speculated that spermidine might improve LPS-induced mitochondrial dysfunction, and consequently, inhibit NLRP3 inflammasome. To this end, we first detect oxygen consumption rates that reflects mitochondrial respiration capacity by high-resolution respirometry with substrates and specific inhibitors. Both the coupled respiration through Complex I (CI) and CII, reflecting oxidative phosphorylation (OXPHOS) level, and uncoupled respiration, providing an estimate of the maximum electron transfer (ET) capacity were significantly enhanced in spermidine-treated macrophages compared with those of LPS and control groups (Fig. 5A). Next, we measured the level of ROS in THP-1 cells with Mito-Sox staining. Compared to the control group, intracellular ROS levels were significantly increased after LPS treatment. Contrastingly, spermidine pretreatment attenuated LPS-induced ROS accumulation in THP-1 macrophages (Fig. 5B). Given that mitochondrial membrane potential is recognized as a key marker of mitochondrial state, we further examined mitochondrial membrane potential by O2k respirometry and JC-1 probes staining. The data showed that the mitochondrial membrane potential of THP-1 macrophages collapsed in response to LPS stimulation, suggested by the increase of fluorescent green JC-1 monomers, while spermidine markedly restored the mitochondrial membrane potential polarization (Fig. 5C, D). Subsequently, we wondered whether spermidine was able to regulate mitochondrial proteins. NADH:ubiquinone oxidoreductase core subunit V2 (NDUFV2) is subunit of complex I and involved in electron transfer. ATP Synthase Subunit Alpha (ATP5A1) is a subunit of mitochondrial ATP synthase, also known as Complex V, produces ATP from ADP. Western blotting analysis found that the expression of NDUFV2 and ATP5A1 were significantly increased by spermidine, suggesting that spermidine may promote mitochondrial biogenesis (Fig. 5E). Inhibition of NLRP3 and pro-IL-1β expression, as well as ROS generation by spermidine were also confirmed in primary peritoneal macrophages (Additional file 1: Fig. S5).

### Spermidine modulates mitochondrial functionality and macrophage inflammatory response through an eIF5A hypusination related pathway

**Fig. 6:**
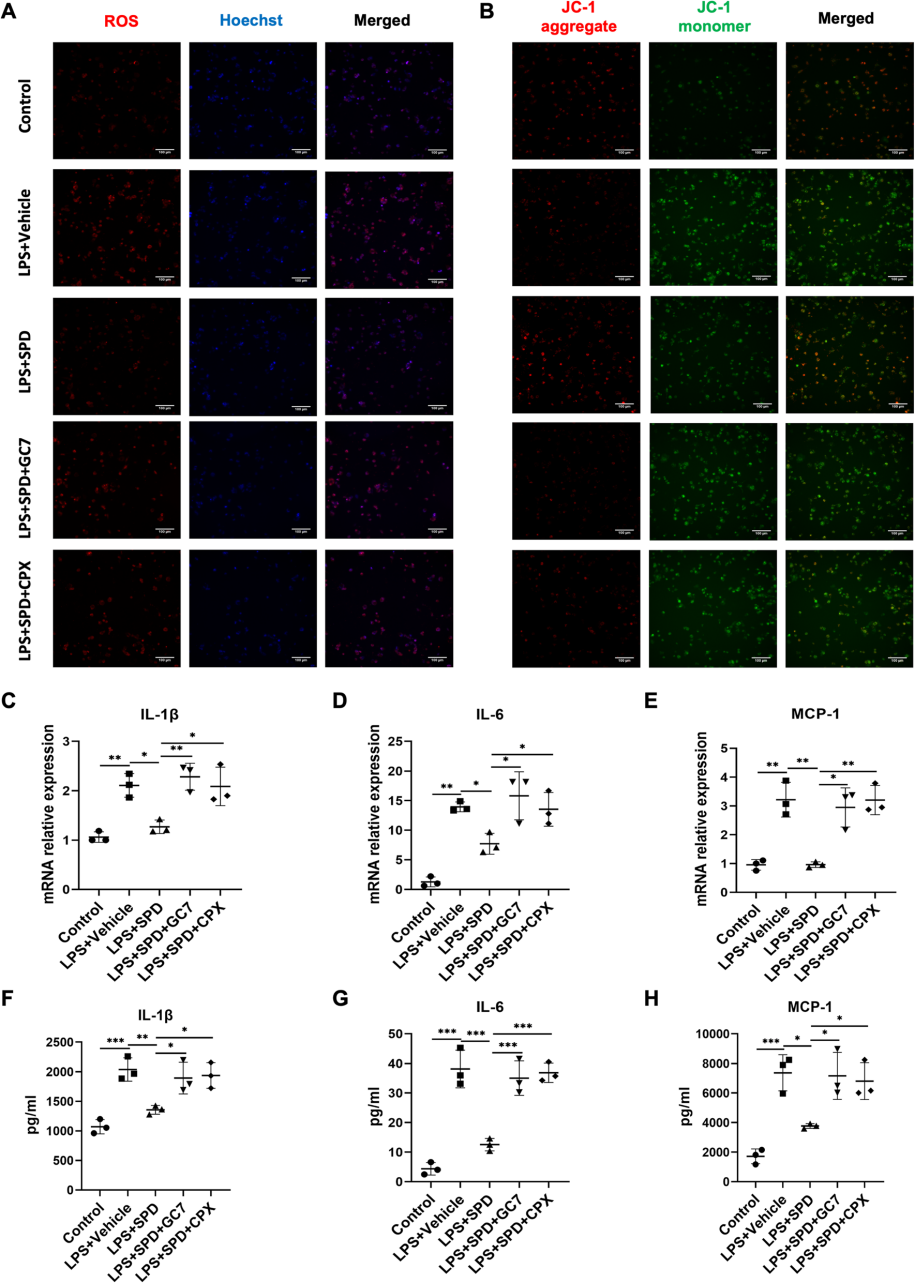
Inhibition of eIF5A^H^ partially abrogated the effects of spermidine on macrophages. Spermidine was administered in the presence or absence of GC7 or CPX, both of which are inhibitors for eIF5A hypusination. **A** ROS (red) and Hoechst (blue) staining. Scale bar, 100 μm. **B** JC-1 staining. Scale bar, 100 μm. Cellular mRNA levels of **C** IL-1β, **D** IL-6, and **E** MCP-1. Concentrations of **F** IL-1β, **G** IL-6, and **H** MCP-1 in the culture supernatants. Experiments were performed in triplicate. Data are expressed as mean ± SEM. Two-way ANOVA followed by post hoc Sidak test. *P < 0.05, **P < 0.01, *** *P* < 0.001

In eukaryotes, spermidine reacts with lysine residue to form hypusine, which is known as hypusination (Park et al. 1981). Notably, this process occurs only in the highly-conserved protein eIF5A and is catalyzed by two key enzymes, deoxyhypusine synthase (DHPS) and deoxyhypusine hydroxylase (DOHH) (Park and Wolff 2018). Serving as a substrate, the abundance of spermidine has a direct influence on the eIF5A hypusination (Diskin et al. 2021). Previous studies have shown that endogenous spermidine involved in macrophage alternative activation through eIF5A hypusination (Puleston et al. 2019). To further clarify the mechanism by which spermidine attenuate macrophage inflammatory responses, the role of eIF5A hypusination was examined with DHPS inhibitor GC7 or DOHH inhibitor ciclopirox (CPX). The data showed that GC7 and CPX partially abolished the regulation of spermidine for mitochondrial membrane potential and ROS (Fig. 6A, B). In addition, both GC7 and CPX treatment potently upregulated the expression of proinflammatory factors in spermidine-primed macrophages (Fig. 6C–H). These results indicated that eIF5A hypusination plays a crucial role for the immunoregulatory capacity of spermidine.

## Discussion

Putrescine, spermidine, and spermine are collectively referred to as polyamines, among which spermidine has emerged as a promising geroprotector (anti-aging supplements) as well as health-promoting agent recently (Madeo et al. 2020). The polyamine metabolism, including cellular biosynthesis and catabolism, keeps balance under the joint action of several key enzymes. During AKI, the polyamine balance is often disrupted by the augmentation of catabolism (Zahedi et al. 2019). Spermine/spermidine-*N*^1^-acetyltransferase (SSAT), a rate-limiting enzyme of polyamine back-conversion, as well as spermine oxidase (SMOX), another important enzyme in polyamine catabolism, are upregulated in kidney tissues in response to different insults, resulting in a significant buildup of putrescine (Zahedi et al. 2003; Wang et al. 2004). In mice, genetically ablation of SSAT and SMOX inhibit tubular injuries and restore kidney function after AKI, suggesting a critical role of endogenous polyamine metabolism in the pathogenesis of kidney injuries (Zahedi et al. 2009, 2010, 2014, 2017). Importantly, exogenous addition of spermidine displays a protective role against kidney injuries induced by a variety of stimuli, including cisplatin, and ischemia–reperfusion injury (Kim 2017a, b; Yoon and Kim 2018). In addition, a recent study revealed that polyamine metabolism is altered in focal segmental glomerulosclerosis and highlighted a crucial role of spermidine in maintaining filtration barrier, suggesting polyamine metabolism is also important in the pathogenesis of chronic kidney diseases (Liang et al. 2020).

In light of the pleiotropic effects of spermidine at both cellular and molecular levels, it is plausible that spermidine may exert its renoprotection by more than one mechanism (Madeo et al. 2018; Pegg 2016). Increasing body of evidence have implicated the immunoregulatory function as an important mechanism in the health-improving action of spermidine (Proietti et al. 2020). Generally, spermidine displays an immunomodulatory property for a wide range of immune cells. For instance, spermidine was found to modulate CD4^+^ T cell differentiation and preferentially prime naïve T cells to regulatory T cells (Puleston et al. 2021). Spermidine can induce immunoregulatory dendritic cells by activating Src kinase and consequently phosphorylating indoleamine 2,3-dioxygenase 1 (Mondanelli et al. 2017). Furthermore, spermidine has been proved to protect against experimental autoimmune encephalomyelitis in mice by inducing immunosuppressive macrophages (Yang et al. 2016). In line with the protective role of spermidine for autoimmune disease, Li et al. found that spermidine could also alleviate inflammatory bowel disease in mice via eliciting anti-inflammatory phenotype in macrophages through mtROS-AMPK-Hif-1α axis and autophagy induction (Liu et al. 2020a). Consistently, we found that the renoprotective effect of spermidine in response to LPS treatment is, at least in part, dependent on macrophages. In support of this, pharmacologically depleting macrophages, to a great extent, abolished spermidine-mediated therapeutic effect for renal injuries. However, it is notable that spermidine might also exert its renoprotective effect through other immune cells, which warrants further studies.

In subsequent assays, we demonstrated that spermidine inhibits macrophage NLRP3 inflammasome activation, IL-1β secretion and related NF-κB signaling, resulting in a substantial suppression of proinflammatory capability of macrophages. These results are in line with the current notion that interventions targeting macrophages to favor an immunoregulatory and tissue-repairing switching could be a potential and more specific therapeutic modality.

In macrophages, metabolic reprogramming between glycolysis and OXPHOS supports the phenotype switching between classical and alternative activation (Huang et al. 2014; Covarrubias et al. 2016). Specifically, alternatively activated macrophages enhance OXPHOS as their predominant energy-producing pathway, while classically activated macrophages upregulate glycolysis instead (Huang et al. 2016). OXPHOS takes place in mitochondria that is the center of energy metabolism and serves as a potential treatment target. Spermidine have been shown to improve mitochondrial health and functionality in different pathophysiologic settings (Eisenberg et al. 2016; Fan et al. 2017). Consistent with previous reports (Liu et al. 2020a), we confirmed a positive role for spermidine in restoring mitochondria fitness in macrophages. Interestingly, with specific inhibitors, we revealed a divergent effect of spermidine on the different part of electron transport chain (ETC). Importantly, we further found that spermidine augments the expression of two mitochondrial proteins participated in ETC. However, the precise mechanism by which spermidine promotes the expression of both proteins remain elusive. It is known that macrophage reprogram their metabolism to regulate mtROS for diverse immune functions (Mehta et al. 2017). mtROS can promote proinflammatory responses in activated macrophages. Blocking ROS, by mitochondrial targeted antioxidant or non-selective inhibitor, results in reduced proinflammatory capacity in macrophages (Kelly et al. 2015; Mills et al. 2016).

**Fig. 7:**
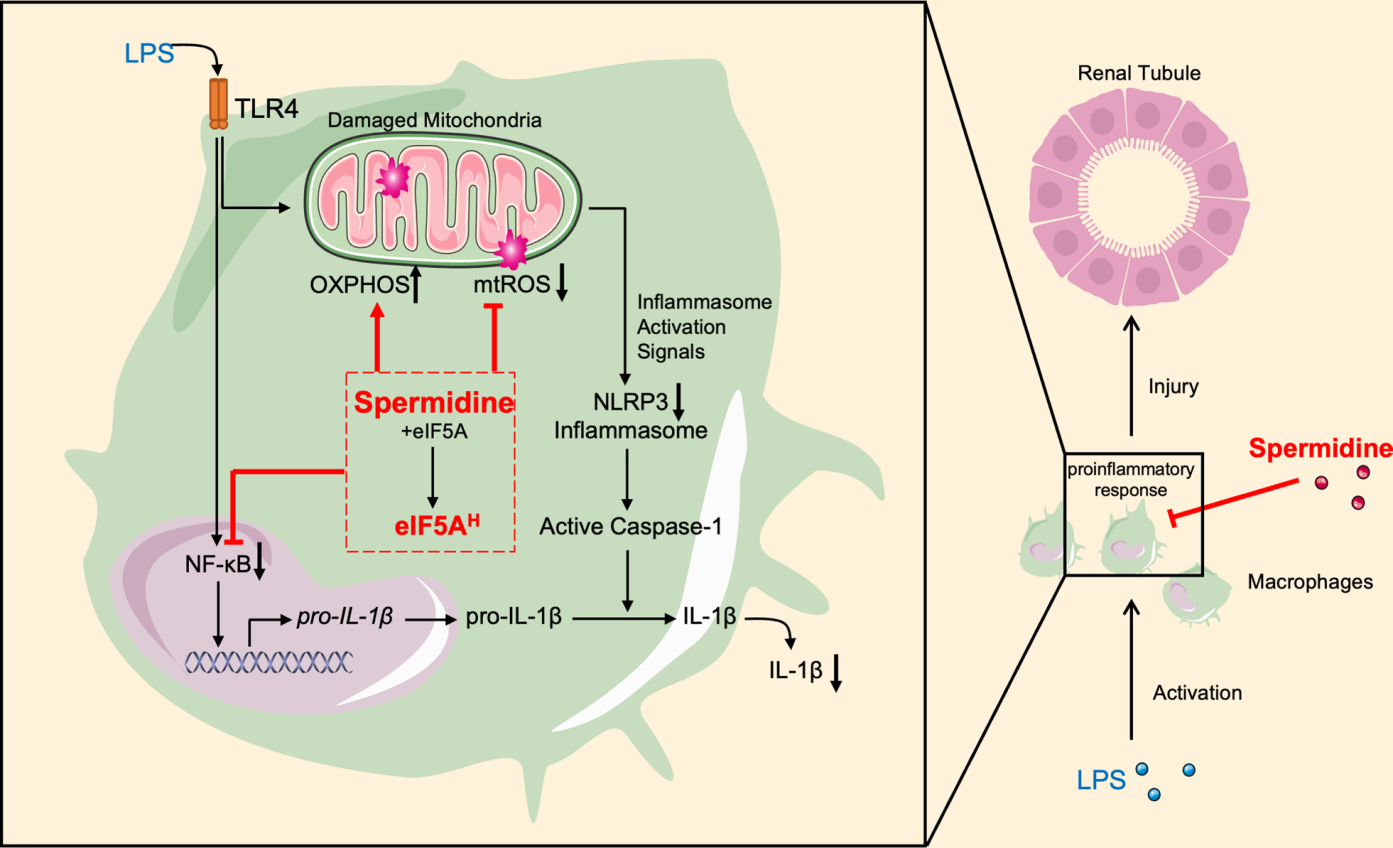
Schematic overview of the protection of spermidine against sepsis-induced AKI. Spermidine protects against AKI by inhibiting macrophage inflammatory responses. LPS activated NLRP3 inflammasome and thereby promoted IL-1β production and secretion in macrophages. In contrast, spermidine downregulates NLRP3 inflammasome activation and IL-1β production in macrophages. Mechanically, spermidine enhances oxidative phosphorylation (OXPHOS) and reduces mitochondrial ROS (mtROS) overload, which contribute to the NLRP3 inhibition. Importantly, eIF5A hypusination can mediate the effects of spermidine on macrophages

Hypusination of eIF5A (eIF5A^H^) is of crucial importance for the translation elongation and termination of select mRNA subsets and regulates both innate and adaptive immunity (Park and Wolff 2018). Zhang et al. found that eIF5A hypusination maintains B cell function via promoting TFEB expression and autophagy (Zhang et al. 2019). Furthermore, it has been reported that hypusinated eIF5A is associated with dendritic cell maturation and T cell differentiation (Puleston et al. 2021). Importantly, Puleston et al. described an indispensable role of eIF5A^H^ in macrophage alternative activation (Puleston et al. 2019). The endogenous spermidine in macrophages maintains tricarboxylic acid cycle and ETC integrity through eIF5A hypusination. Hypusinated eIF5A also promotes OXPHOS by controlling mitochondrial proteins expression and thereby enhances macrophage alternative activation. However, inhibition of eIF5A^H^ did not affect classical activation of macrophage (Puleston et al. 2019). Interestingly, we demonstrated that exogenous supplementation of spermidine could diminish macrophage activation in an eIF5A^H^-dependent pathway. Inhibitors for DHPS as well as DOHH markedly curtailed the effect of spermidine on macrophage IL-1β production and mitochondrial activity (Fig. 7).

It is well-documented that spermidine induces autophagy in a wide spectrum of tissues and cells (Eisenberg et al. 2009; Gupta et al. 2013). Due to the prevalent health-promoting property of autophagy in diverse pathophysiologic settings, spermidine might confer a general positive impact via autophagy directly. A previous study reported that spermidine-mediated alternatively polarization relies on autophagy (Sun et al. 2019). Interestingly, hypusinated eIF5A is required for the expression of autophagy transcription factor TFEB and thereby regulates autophagy (Zhang et al. 2019). In this scenario, it is reasonable to speculate that spermidine-driven eIF5A hypusination is interrelated with autophagy in spermidine-treated macrophage. However, to what extent autophagy is dependent on eIF5A hypusination and how eIF5A^H^-autophagy axis contribute to the immunomodulation of spermidine need further investigations.

Recently, epidemiologic studies in human confirmed that dietary spermidine uptake is reversely correlated with cardiovascular, cancer-related and all cause mortalities (Eisenberg et al. 2016; Kiechl et al. 2018; Madeo et al. 2018). However, more prospective clinical trials are warranted to examine the therapeutic potential of spermidine in human. The treatment effect of spermidine has been under intensive investigation in animal models. A critical challenge in translating experimental findings to clinical applications will be the conversion of animal doses to human equivalent doses. For example, 50 mg/kg of spermidine applied in the present study is approximately, based on body surface area, equivalent to 4 mg/kg for human, which is much more than the dietary uptake of spermidine (Munoz-Esparza et al. 2019). Although spermidine can be synthesized in cells and commonly present in food, potential side effects of high dose supplementation should be tested.

## Conclusions

We have demonstrated that spermidine administration protects against sepsis-induced AKI in mice and that macrophages serve as an essential mediator in this protective effect. We further determined the underlying mechanism by which spermidine modulates macrophage activity. Our study identifies spermidine as a promising pharmacologic approach to prevent AKI. Further investigations on the strategies to optimize the therapeutic efficacy of spermidine are warranted.

## Supplementary Information

**Additional file 1: Figure S1.** Time and dose–response study of spermidine for mice model. **Figure S2.** Spermidine facilitates kidney recovery. **Figure S3.** Effect of clodronate liposome on macrophage. **Figure S4.** Macrophage infiltration detection by immunohistochemistry. **Figure S5.** Spermidine inhibits NLRP3 inflammasome activation and ROS generation in primary peritoneal macrophages.

**Additional file 2: Table S1.** Primers used for qPCR analysis.

## Data Availability

The raw data supporting the conclusion of this article will be made available by the authors, upon reasonable request.

## Abbreviations

AKI: Acute kidney injury
eIF5A: Eukaryotic initiation factor 5A
Cr: Serum creatinine
BUN: Blood urea nitrogen
HE: Hematoxylin and eosin
TUNEL: TdT mediated dUTP nick end labeling
FBS: Fetal bovine serum
ELISA: Enzyme-linked immunosorbent assay
qPCR: Quantitative polymerase chain reaction
ROS: Reactive oxygen species
MCP-1: Monocyte chemoattractant protein-1
TNF-α: Tumor necrosis factor-α
IL-1β: Interleukin-1β
IL-6: Interleukin-6
ICAM-1: Intercellular cell adhesion molecule-1
CL: Clodronate liposomes
EL: Empty liposomes
NLRP3: NOD-like receptor protein 3
KIM-1: Kidney injury molecule-1
NGAL: Neutrophil gelatinase-associated lipocalin
DHPS: Deoxyhypusine synthase
DOHH: Deoxyhypusine hydroxylase
CPX: Ciclopirox
SSAT: Spermine/spermidine-*N*^1^-acetyltransferase
SMOX: Spermine oxidase
ETC: Electron transport chain
OXPHOS: Oxidative phosphorylation
NDUFV2: NADH:ubiquinone oxidoreductase core subunit V2
ATP5A1: ATP Synthase Subunit Alpha
CXCL-1: Chemokine (C-X-C motif) ligand 1
