# Supplementary material for: Spermidine protects against acute kidney injury by modulating macrophage NLRP3 inflammasome activation and mitochondrial respiration in an eIF5A hypusination-related pathway
Source: Mol Med. 2022 Sep 4;28:103. doi: 10.1186/s10020-022-00533-1 (PMC9441050; doi:10.1186/s10020-022-00533-1)
Supplement: Supplementary file 1 — Additional file 1: Figure S1. Time and dose–response study of spermidine for mice model. Figure S2. Spermidine facilitates kidney recovery. Figure S3. Effect of clodronate liposome on macrophage. Figure S4. Macrophage infiltration detection by immunohistochemistry. Figure S5. Spermidine inhibits NLRP3 inflammasome activation and ROS generation in primary peritoneal macrophages. [file 10020_2022_533_MOESM1_ESM.pdf]

Fig. S1

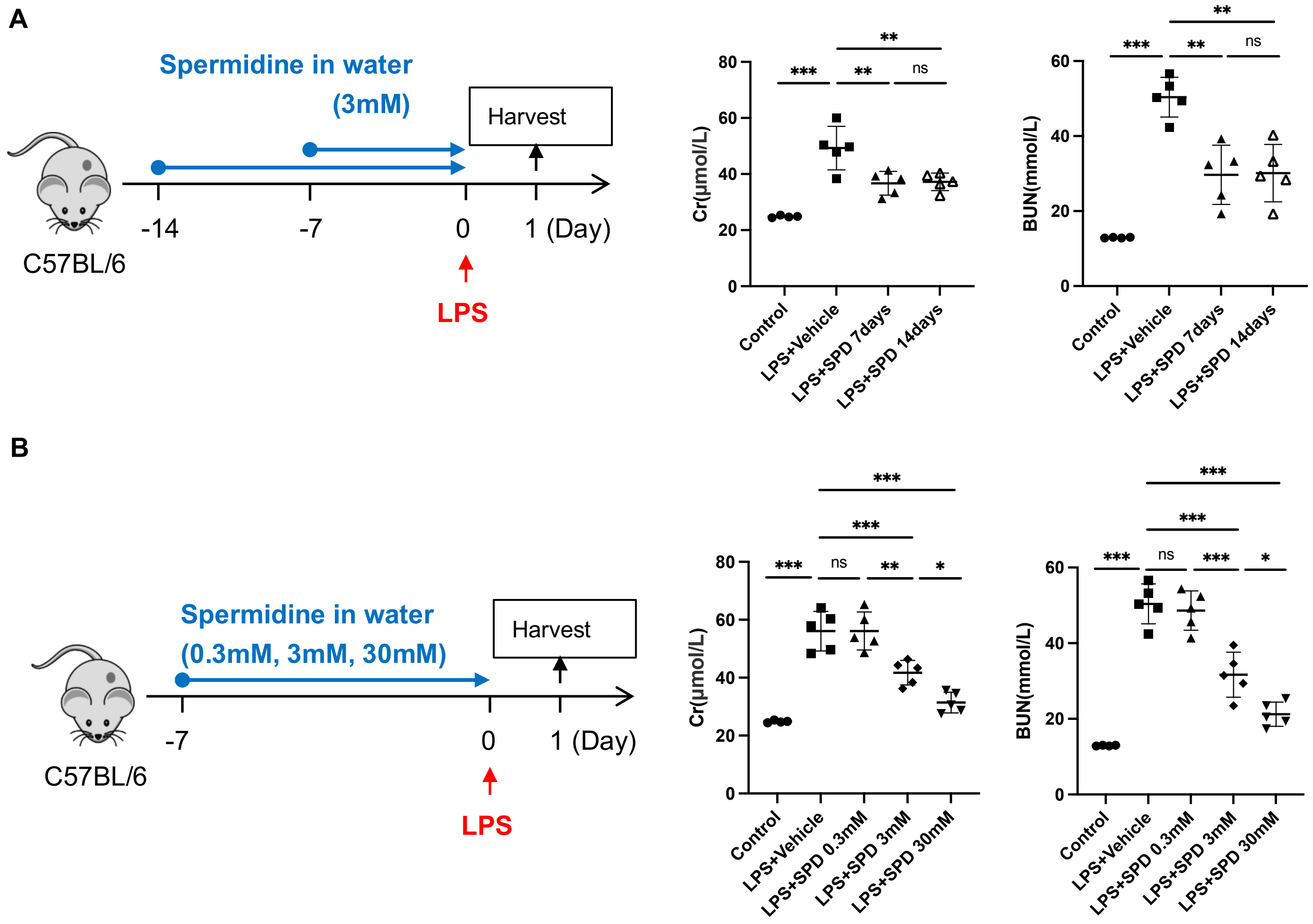

Fig. S1. Time and dose-response study of spermidine for mice model. (A) Spermidine was administered into drinking water (3 mM) for 1 week or 2 weeks before sepsis induction. Mice were subjected to LPS intraperitoneally at a dose of 10 mg/kg. Twenty-four hours after LPS injection, mice were sacrificed for blood and kidney samples. Levels of serum creatinine (Cr) and blood urea nitrogen (BUN) were detected. (B) Spermidine was supplemented via drinking water at different doses (0.3mM, 3 mM, 30 mM) for 1 week before LPS injection. Twenty-four hours after LPS injection, mice were sacrificed for blood and kidney samples. Levels of serum creatinine and blood urea nitrogen were determined. n=4 or 5 mice per group. Data are expressed as mean ± SEM. One-way ANOVA followed by post hoc Sidak test. * $P < 0.05$, ** $P < 0.01$, *** $P < 0.001$.

Fig. S2

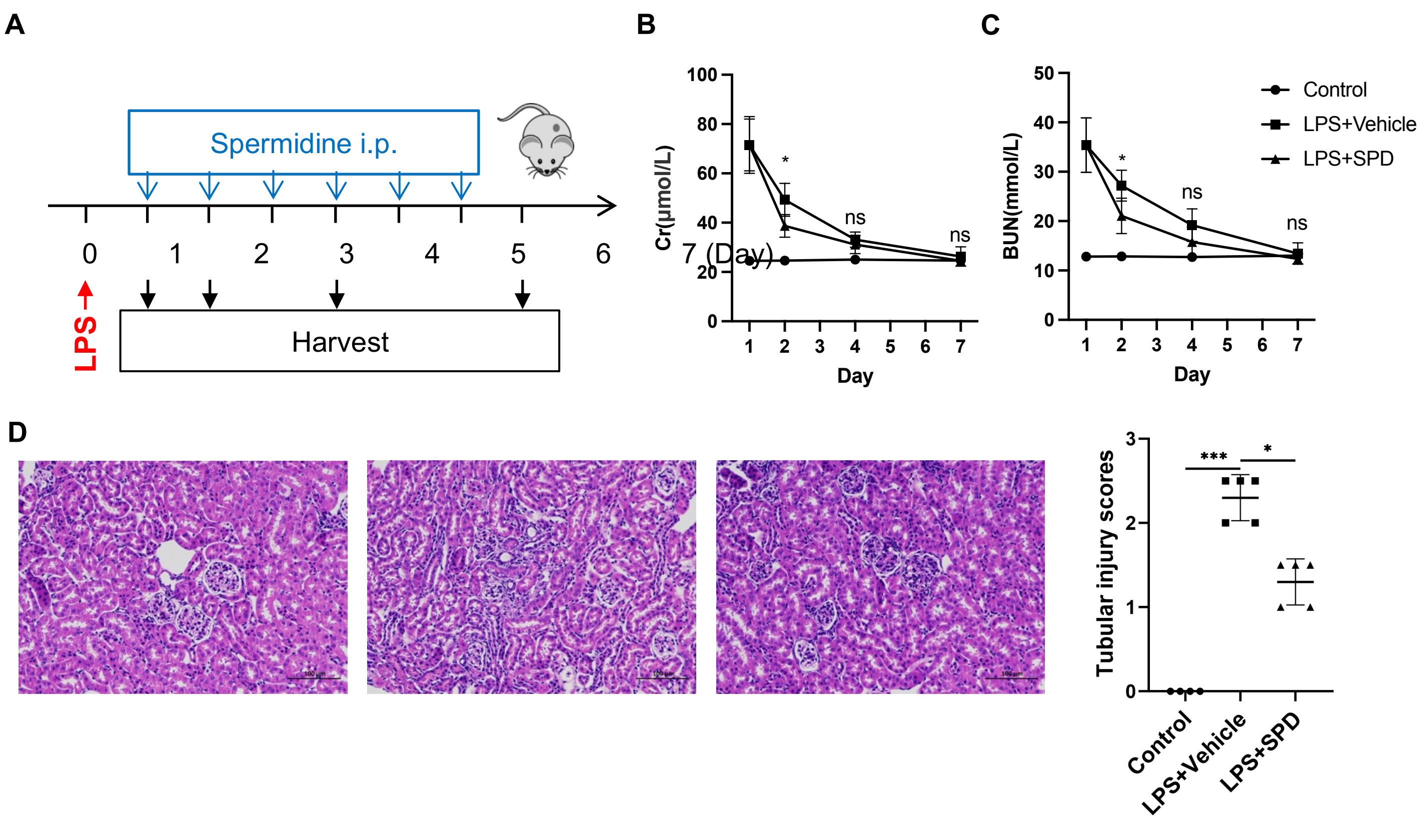

Fig. S2. Spermidine facilitates kidney recovery. (A) Spermidine (50mg/kg) was administered intraperitoneally (i.p.) daily 24 hours after LPS injection. Levels of (B) serum creatinine (Cr) and (C) blood urea nitrogen (BUN) were detected. * $P < 0.05$ LPS+Vehicle *vs* LPS+SPD. Control, n=4 per time point; other groups, n=5 per time point. (D) Tubular injury score was evaluated on day 2 after LPS injection. Scale bar, 100 μm. Data are expressed as mean ± SEM. One-way ANOVA followed by post hoc Sidak test. * $P < 0.05$, ** $P < 0.01$, *** $P < 0.001$.

Fig. S3

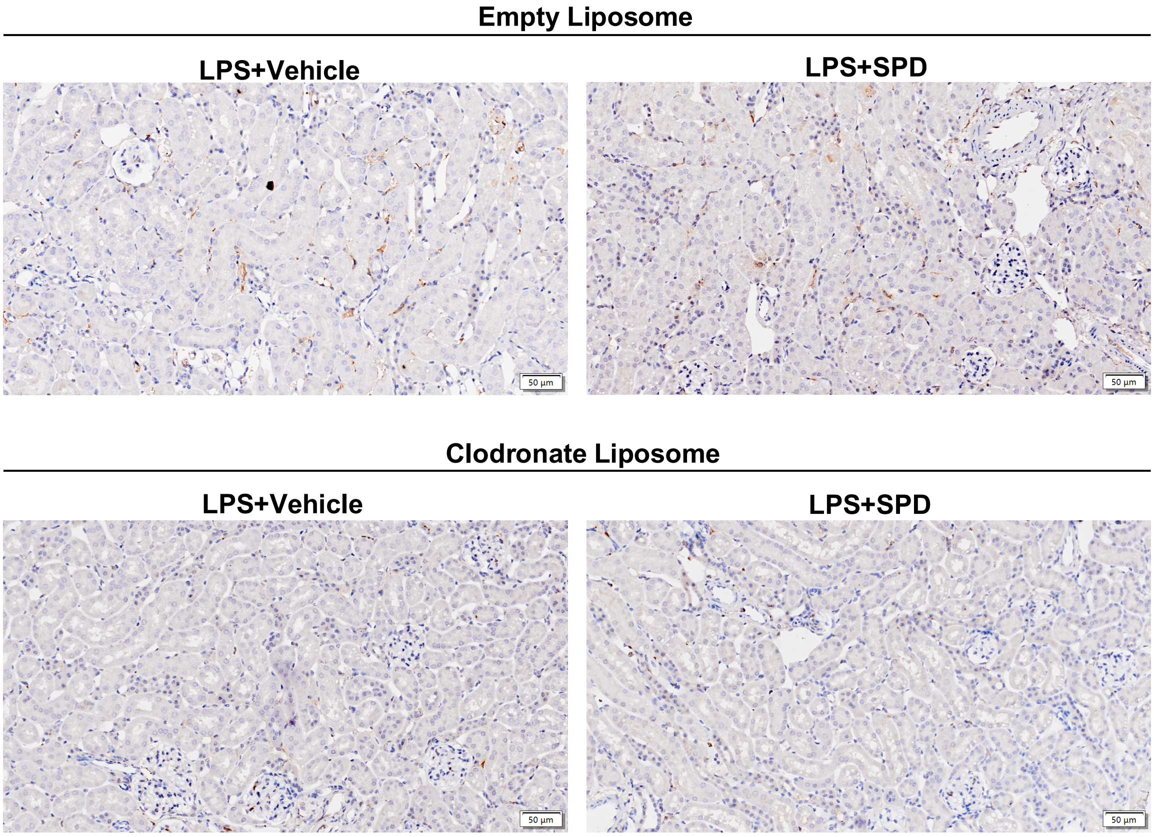

Fig. S3. Effect of clodronate liposome on macrophage. The depletion efficacy of macrophage by clodronate liposome was confirmed through immunohistochemical detection of F4/80 in kidney tissues. Scale bar, 50 μm.

Fig. S4

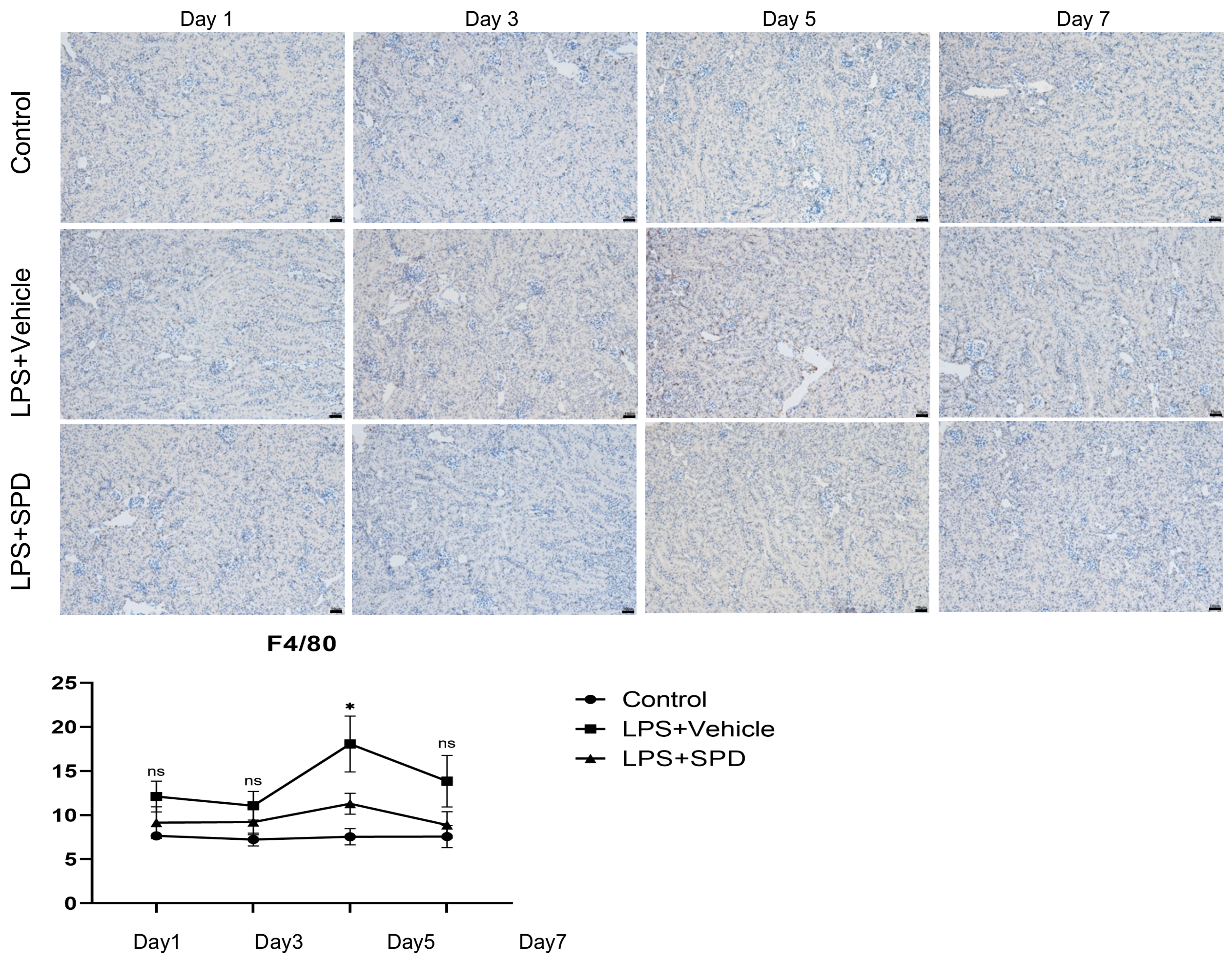

Fig. S4. Macrophage infiltration detection by Immunohistochemistry. Immunohistochemical staining of F4/80 in kidney tissues. The average number of positive cells was analyzed. Scale bar, 10 µm. Control, n=4 per time point; other groups, n=5 per time point. Data are expressed as mean ± SEM. One-way ANOVA followed by post hoc Sidak test. * $P < 0.05$ LPS+Vehicle *vs* LPS+SPD.

Fig. S5

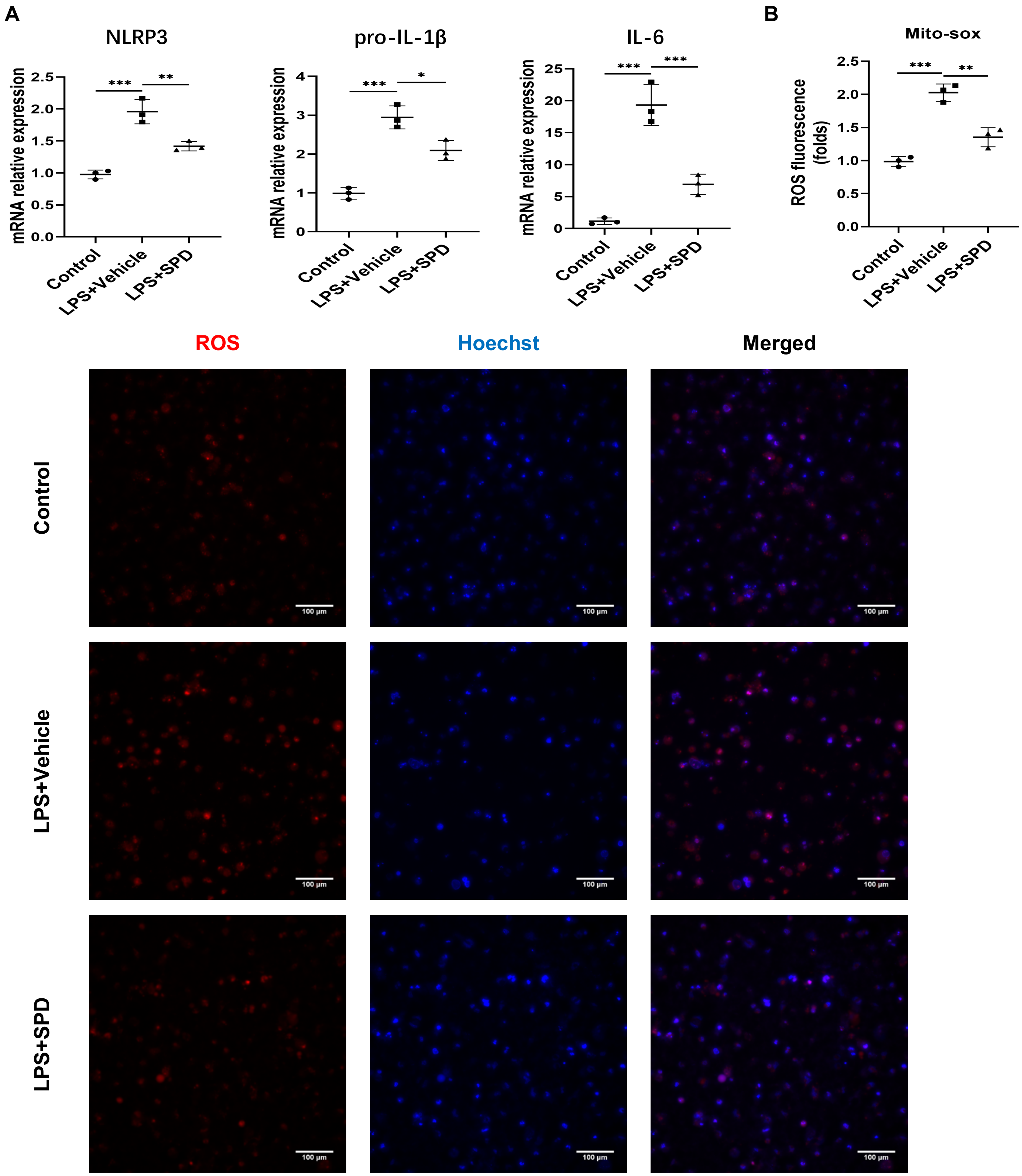

Fig. S5. Spermidine inhibits NLRP3 inflammasome activation and ROS generation in primary peritoneal macrophages. Peritoneal macrophages were pretreated with spermidine (50μM) for 1h and then stimulated by LPS (200 ng/ml) for 24h. (A) NLRP3, pro-IL-1β and IL-6 mRNA expression were detected by qPCR. (B) ROS generation was examined by MitoSox (red) staining. Scale bar, 100 μm. Fluorescence intensity was calculated using Image J software. Experiments were performed in triplicate. Data are expressed as mean ± SEM. One-way ANOVA followed by post hoc Sidak test. * $P < 0.05$, ** $P < 0.01$, *** $P < 0.001$.
