# Supplementary material for: Spermidine protects against acute kidney injury by modulating macrophage NLRP3 inflammasome activation and mitochondrial respiration in an eIF5A hypusination-related pathway
Source: Mol Med. 2022 Sep 4;28:103. doi: 10.1186/s10020-022-00533-1 (PMC9441050; doi:10.1186/s10020-022-00533-1)
Supplement: Supplementary file 2 — Additional file 2: Table S1. Primers used for qPCR analysis. [file 10020_2022_533_MOESM2_ESM.docx]

**Table S1.** Primers used for qPCR analysis

| Species | Genes | Forward sequence | Reverse sequence |
| --- | --- | --- | --- |
| Mouse | KIM-1 | AGCCGCAGAAAAACCCTACT | AACCACGCTTAGAGATGCTGA |
|  | NGAL | GGCCAGTTCACTCTGGGAAA | TGGCGAACTGGTTGTAGTCC |
|  | TNF-α | CAGTGGAGCAGGTGAAGAGT | AGATGTCAAATTCATTCATGGCCT |
|  | IL-1β | GAAATGCCACCTTTTGACAGTGATG | TTCTCCACAGCCACAATGAGT |
|  | IL-6 | CTTCTTGGGACTGATGCTGGT | CTCTGTGAAGTCTCCTCTCCG |
|  | MCP-1 | CCTGCTGCTACTCATTCACCA | ATTCCTTCTTGGGGTCAGCA |
|  | ICAM-1 | TTCTTTTGCTCTGCCGCTCT | CCTCTTGCCAGGTCCAGTTC |
|  | β-actin | GGCTGTATTCCCCTCCATCG | CCAGTTGGTAACAATGCCATGT |
| Human | TNF-α | GAGGCCAAGCCCTGGTATG | CGGGCCGATTGATCTCAGC |
|  | IL-1β | AGCTGGAGAGTGTAGATCCCAA | ACGGGCATGTTTTCTGCTTG |
|  | IL-6 | CAATGAGGAGACTTGCCTGGT | GCAGGAACTGGATCAGGACT |
|  | MCP-1 | CTCAGCCAGATGCAATCAATG | CTTCTTTGGGACACTTGCTGC |
|  | NLRP3 | TATGTGGGGGAGAATGCCTTG | ACCGAGGACAAAGCTGAACAA |
|  | CXCL-1 | TTGCCTCAATCCTGCATCCC | GTTGGATTTGTCACTGTTCAGCAT |
|  | CXCL-2 | TTGTCTCAACCCCGCATCG | CAGTTGGATTTGCCATTTTTCAG |
|  | β-actin | CATGTACGTTGCTATCCAGGC | CTCCTTAATGTCACGCACGAT |
